# By counteracting gravity, triceps surae sets both kinematics and kinetics of gait

**DOI:** 10.1002/phy2.229

**Published:** 2014-02-10

**Authors:** Jean‐Louis Honeine, Marco Schieppati, Oliver Gagey, Manh‐Cuong Do

**Affiliations:** 1 Complexité, Innovation et Activité Motrices et Sportive laboratory, Sport‐Science Faculty, University Paris‐Sud, Orsay, F‐91405, France; 2 Centro Studi Attività Motorie laboratory, Salvatore Maugeri Foundation (IRCCS) and Department of Public Health, Experimental and Forensic Medicine, University of Pavia, Pavia, I‐27100, Italy; 3 Department of Orthopaedics, Faculty of Medicine, University Paris‐Sud, Le Kremlin‐Bicêtre, F‐94276, France

**Keywords:** Step duration, step length, triceps surae

## Abstract

In the single‐stance phase of gait, gravity acting on the center of mass (CoM) causes a disequilibrium torque, which generates propulsive force. Triceps surae activity resists gravity by restraining forward tibial rotation thereby tuning CoM momentum. We hypothesized that time and amplitude modulation of triceps surae activity determines the kinematics (step length and cadence) and kinetics of gait. Nineteen young subjects participated in two experiments. In the gait initiation (GI) protocol, subjects deliberately initiated walking at different velocities for the same step length. In the balance‐recovery (BR) protocol, subjects executed steps of different length after being unexpectedly released from an inclined posture. Ground reaction force was recorded by a large force platform and electromyography of soleus, gastrocnemius medialis and lateralis, and tibialis anterior muscles was collected by wireless surface electrodes. In both protocols, the duration of triceps activity was highly correlated with single‐stance duration (GI,* R*^2^ = 0.68; BR,* R*^2^ = 0.91). In turn, step length was highly correlated with single‐stance duration (BR,* R*^2^ = 0.70). Control of CoM momentum was obtained by decelerating the CoM fall via modulation of amplitude of triceps activity. By modulation of triceps activity, the central nervous system (CNS) varied the position of CoM with respect to the center of pressure (CoP). The CoM‐CoP gap in the sagittal plane was determinant for setting the disequilibrium torque and thus walking velocity. Thus, by controlling the gap, CNS‐modified walking velocity (GI,* R*^2^ = 0.86; BR,* R*^2^ = 0.92). This study is the first to highlight that by merely counteracting gravity, triceps activity sets the kinematics and kinetics of gait. It also provides evidence that the surge in triceps activity during fast walking is due to the increased requirement of braking the fall of CoM in late stance in order to perform a smoother step‐to‐step transition.

## Abstract (precis)

The article shows that by vertically controlling the downward fall of the center of mass the central nervous system is capable of setting global gait kinematics and kinetics. The control is done via temporal modulation of triceps surae activity. The article also shows that increasing the amplitude of triceps activity in late stance brakes the fall of the center of mass providing smooth conditions for the subsequent step‐to‐step execution.

## Introduction

In daily life, we walk at different velocities. Walking necessitates proper orchestration of lower limb muscle activity by the central nervous system (CNS) in order to set specific kinetic and kinematic parameters. Kinetically, locomotion requires the application of a propulsive force to accelerate the body and achieve a certain walking velocity. Kinematic variables are strictly linked together by the equation: *V* = *L* × *C*, where *V* is walking velocity, *L* is step length, and *C* is cadence (Nilsson et al. 1985; Brenière 1996; Bertram and Ruina 2001). Many studies have investigated the relationship between the three variables (Alexander 1984; Nilsson et al. 1985; Stoquart et al. 2008; Ivanenko et al. 2011; Leurs et al. 2011). These studies, mostly conducted on a treadmill, proved that all combinations between walking velocity, step length, and cadence are possible.

Bipedal gait is a particular form of locomotion, characterized by a succession of single‐ and double‐stance phases. During single stance, gravity causes the body's center of mass (CoM) to fall and rotate around the stance foot where the ground reaction force (GRF) is applied. Cavagna et al. (1976) showed that the downward kinetic energy of the CoM is transformed into forward propulsive energy during walking. In a previous study, we showed how gravity acting on the CoM generates a disequilibrium torque, which is responsible for propelling the body (Honeine et al. 2013). The disequilibrium torque was the product of the anteroposterior (AP) distance between CoM and center of foot pressure (CoP) by the vertical force acting on the CoM. In that study, we also provided evidence, by monitoring the triceps electromyography (EMG) activity when subjects initiated gait with and without a load. Loading the subjects necessarily increased propulsive force but did not alter the amplitude of the triceps EMG. We thus concluded that the role of the triceps surae during gait is to maintain dynamic postural equilibrium during single stance and is not directly responsible in the generation of propulsive force. Nonetheless, the amplitude of triceps surae activity of the stance leg covaried with walking velocity during single stance (Winter 1983; Hof et al. 2002; Den Otter et al. 2004). In the present study, we investigated the mode through which the time‐course of triceps activity can ultimately produce different walking velocities. Furthermore, in order to advance knowledge of the mechanisms underpinning the covariation between walking speed and triceps amplitude, we investigated whether the rise in triceps activity in fast walking is imposed by the need of additional braking action to counteract the fall of CoM.

We took advantage of the inverted pendulum mechanics in order to understand how modulation of the amplitude and duration of triceps surae activity sets walking velocity. In a passive inverted pendulum, CoM momentum is generated solely by the pull of gravity. Nonetheless, the CNS controls CoM momentum (Popovic et al. 2004; Herr and Popovic 2008; Neptune and McGowan 2011), as triceps surae restrains tibial rotation (Sutherland et al. 1980) during single stance. We hypothesized that modulation in amplitude and duration of triceps activity determines both kinematics and kinetics of gait. More precisely, we postulated that control over the amplitude of triceps activity allows the CNS to adjust CoM momentum, while the duration of triceps activity determines the duration of the single stance. By controlling CoM momentum and single‐stance duration, the CNS should set how far the body travels away from the stance foot before the foot contact (FC) of the swing leg, that is, it would determine step length. In the process, the distance of CoM with respect to CoP, or the CoM‐CoP gap, will be set. This, in turn, determines the amplitude of the disequilibrium torque, which generates the propulsive force and adjusts walking velocity.

In order to test our hypothesis, we exploited two complementary protocols, in which certain variables can be isolated. In the first experiment, we investigated how triceps controls walking velocity when subjects walk at a fixed step length. In the second experiment, we examined how triceps defines step length. Combining the results of both experiments would allow understanding how the pattern of triceps surae activity sets velocity in all the kinematic combinations of gait.

In the first experiment, we opted for the gait initiation protocol (GI). In GI, when subjects start walking spontaneously, they perform each time a step of a rather constant length (Brenière and Do 1991; MacDougall and Moore 2005). According to the equation *V* = *L* × *C*, if subjects are instructed to vary walking velocity without changing step length then the duration of the single stance has to be modulated. We expect that to walk slowly, the CoM momentum should decrease while the duration of single stance should increase. The opposite should occur when walking faster. In the preparatory phase of GI, the amplitude of the CoM momentum is controlled by the tibialis anterior (TA) activity (Nardone and Schieppati 1988; Lepers and Brenière 1995). During the following single‐stance phase, the amplitude of the triceps activity should be modulated in order to fine‐tune the CoM momentum and react to the CoM fall.

In the second experiment, designed to test how step length is determined, we resorted to the balance‐recovery protocol (BR). In BR, subjects are positioned at an inclined body‐axis orientation and then released. When released, the fall provokes an automatic step, the length of which is determined by the angle of inclination (Do et al. 1982; Pai and Patton 1997; Aftab et al. 2012). Asking subjects to vary step length, without changing the initial inclination, should modulate the duration of triceps surae activity accordingly. Reducing the duration of triceps activity should cause earlier contact with the ground resulting in a short step. The opposite should occur when performing a longer step. Notably, the CoM momentum of the body does not change prior to single stance when subjects are asked to recover balance with different step lengths (Do et al. 1982). This should allow us to investigate how triceps modifies CoM momentum without the influence of events occurring before the onset of the single‐stance phase.

## Methods

Nineteen healthy volunteers (nine females and 10 males) took part in the experiments after giving written informed consent as required by the Helsinki Declaration and the local Ethics committee. Their mean age, body mass (BM), and height were 25 years (range 20–29), 72 kg (48–92), and 1.76 m (1.61–1.83), respectively. Eleven subjects participated in the GI protocol. Eleven subjects performed the BR experiment. Three subjects performed both experiments. Two different groups were used for each of the two experiments because the results of each protocol were not to be directly confronted.

### Experimental setup

A large force platform (0.90 m wide, 1.80 m long, AMTI, Watertown, MA) was used to record GRF and moments. The platform was embedded in the ground and placed at 0.5 m from the wall. Subjects stood on the platform so that the GRF of the first step could be recorded. The entire walkway was long enough for the subjects to carry out several steps. Surface EMG activity was recorded from the soleus (SOL), gastrocnemius medialis (GM), gastrocnemius lateralis (GL), and TA muscles of both legs using bipolar Ag‐AgCl electrodes (8 mm diameter, 20 mm interelectrode distance). Electrodes were placed according to the SENIAM protocol (Merletti and Hermens 2000) after preparing the skin to minimize impedance. EMG activity was on‐site preamplified (×1000, Zero‐Wire, Aurion, Milan, Italy), wirelessly sent to a PC and band‐pass filtered (10–500 Hz). Force platform and EMG data were digitized at a sampling frequency of 1000 Hz on the same A/D converter card and saved for off‐line analysis. A Matlab 2008b (Mathworks, Natick, MA) routine was used for processing the data.

For the BR protocol, an electromechanical device composed of a load cell (S‐Beam; Vishay Celtron, Malvern, PA) coupled to an electromagnet was attached via a sliding joint to a vertical shaft fixed to the wall (Do et al. 1982). An abdominal belt was used to attach the subject to the device by means of a steel cable. This allowed subjects to stand quietly in a forward‐inclined position. The sliding joint permitted to set the cable horizontally. The cable was released by operating on the electromagnet. The load cell was used to identify the time of the release of the cable. It also helped measure the body inclination of the subjects, where inclination = tan^−1^ (force of load cell/Ver GRF). To ensure that the cable was kept horizontal, the subjects' bodyweight was measured under quiet stance. A change in this measure while inclined indicated that the tension in the cable had a vertical component (i.e., the cable was not horizontal).

### Protocols

Subjects stood barefoot on the platform, looking straight ahead. The contour of the feet was drawn with a chalk on the platform so that the subjects always stood in the same position throughout the experiment.

For GI, the subjects stood erect on the platform until they were instructed to initiate gait following a verbal go‐signal. Then, they continued walking until the end of the walkway. They were told not to start walking in a reaction‐time mode, but when they felt ready. This usually occurred within 2 sec from the go‐signal. The subjects performed three sets of walking tasks composed of 10 trials each. In the first task, the subjects started walking at their usual velocity (normal condition). Then, they were asked to walk slow or to walk fast. The slow and fast tasks were performed in random order across the subjects.

For BR, the subjects were inclined forward and stood as relaxed as possible. Body inclination with respect to the vertical was kept at 15° and monitored by means of the feedback from the force platform and the force transducer on the cable. Upon release, the subjects continued walking until the end of the walkway. The time of release was unknown to them. The subjects performed three sets of walking tasks composed of 10 trials each. In the first task, subjects were given no instruction about step length. Then, they performed short and long steps. The order of the short‐step and long‐step tasks was randomized across the subjects.

Subjects initially performed six blank trials for both GI and BR to get accustomed to the protocols. During the blank tests, the preferential leading foot of each subject was detected so that subjects would later initiate gait or recover from fall using the same leading leg throughout the experiment. On average, each of the experiments (GI or BR) lasted 45–60 min.

### Ground reaction force

The platform measured the GRFs and moments in the AP, mediolateral (ML), and vertical (Ver) directions. From these, the CoP coordinates were computed, according to an established procedure (Brenière and Do 1991; McIlroy and Maki 1999). The time of foot off (FO), that is, the onset of the single‐stance phase of gait, was the instant when the ML CoP moved under the stance foot. The time of foot contact was the instant when the AP CoP position shifted abruptly forward. The procedure of timing the FO and FC by means of the force platform output was previously validated with respect to foot‐switch data (Caderby et al. 2013). The CoM momentum is calculated as: Momentum = *m* × *v*, where *m* is the mass of the subject and *v* is the velocity of CoM. CoM velocity was obtained by time‐integrating CoM acceleration. CoM AP acceleration was calculated as AP GRF/BM, and CoM vertical acceleration as (Ver GRF − BW)/BM, where BW and BM are body weight and body mass, respectively. BW and BM were measured by the force platform when subjects were standing still in upright posture. CoM position was obtained by time‐integrating CoM velocity. In GI, the initial position of CoM in the AP direction was considered to be equal to that of the CoP (Winter 1995). In BR, the subjects were initially inclined, so that the CoM was positioned away from the CoP. To estimate the initial position of CoM, we asked subjects to stand in bipedal erect stance. The horizontal distance between their anterior superior iliac spine and the wall behind them was measured (d1). This was compared with the same distance (d2) measured when subjects were inclined for the task. Hence, the CoM initial AP position was (d2 − d1).

### Disequilibrium torque

During gait, CoM moves up and down while rotating around the CoP in the sagittal plane, and the CoM fall is braked during the single‐stance phase of gait. The displacement of the CoM generates a disequilibrium torque, driven by gravity, as CoM moves beyond the CoP. The disequilibrium torque was calculated as Ver GRF × (CoM − CoP). The difference (CoM − CoP) represents the instantaneous distance (hereafter called the gap) between the AP position of the CoP and the corresponding position of the ground projection of the CoM.

### EMG analysis

**Figure 1.**
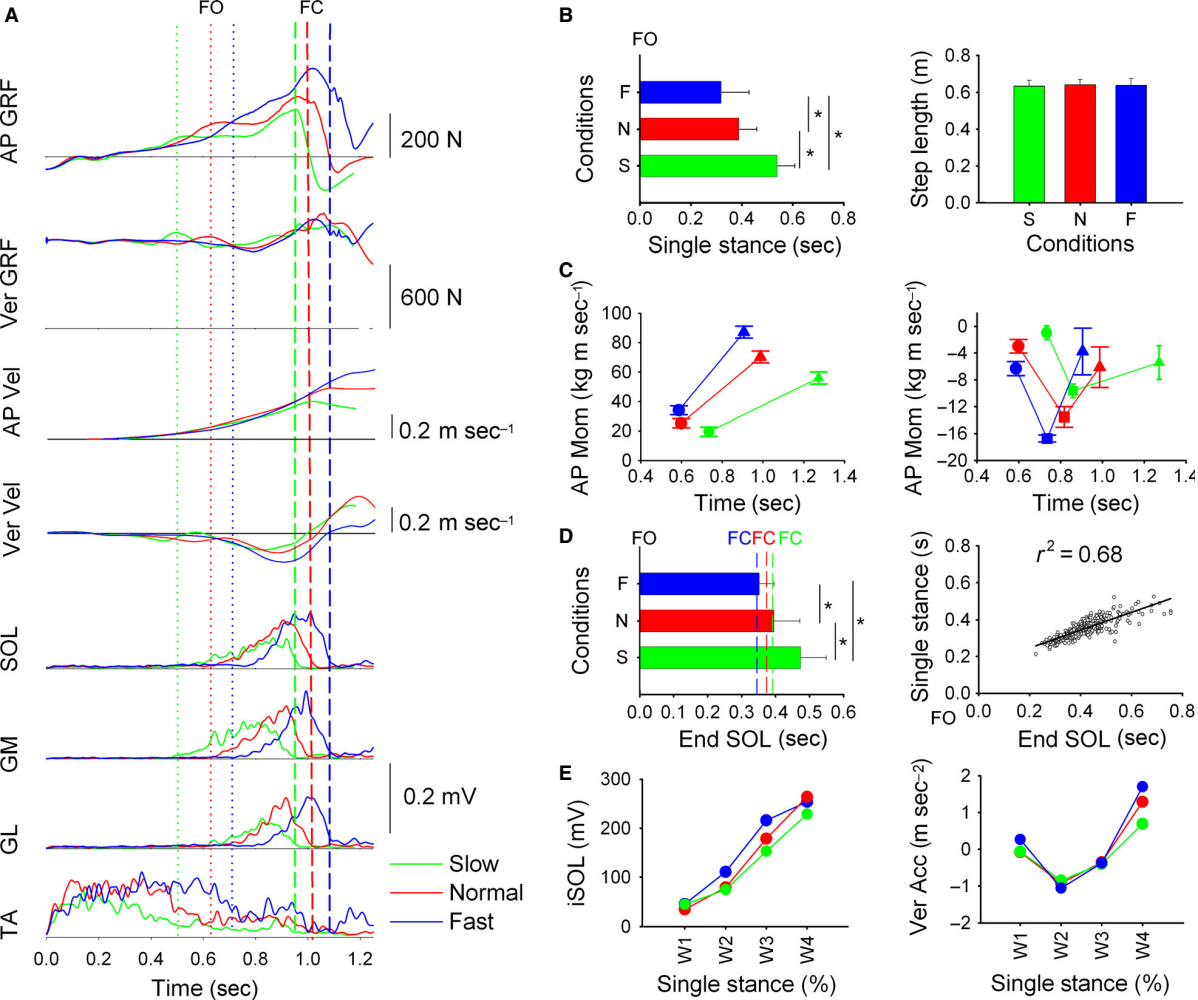
Gait initiation. Green, red, and blue indicate slow, normal, and fast walking velocity, respectively. (A) shows the time‐course of gait initiation variables in a representative subject (each trace is the average of 12 trials). Mechanical variables (AP and Ver GRF and CoM velocity) and the EMG envelopes of SOL, GM, GL, and TA are shown from top to bottom. Vertical dotted lines indicate the instant of foot off (FO), dashed lines the instant of foot contact (FC). In (B, left) the horizontal bars indicate the grand mean ± SD of duration of single stance. In (B, right) the bars depicts the grand mean ± SD of step length. In (C, left) the plot depicts the grand mean ± SD of CoM AP momentum at FO (circles) and FC (triangles). In (C, right) the plot depicts the grand mean ± SD of CoM Ver momentum at FO (circles), minimum value (square) and FC (triangles). In (D, left) the horizontal bars are the grand mean ± SD of the end of activity of the EMG activity of SOL with respect to FO (*t* = 0). The dashed lines indicate the grand mean of the time of FC. In (D, right) the time instant of the end of soleus activity with respect to FO is plotted against the single‐stance duration (all trials collapsed). There is a strict correspondence between the duration of muscle activity and duration of single stance. The linear equation is: *y* = 0.49*x* + 0.14 (E) shows the average values of the level of EMG activity of SOL (left) and the average CoM vertical acceleration (right) measured in the four equal time‐windows, in which the single stance was divided. The mean level of EMG increases with the progression of the single stance and increases in parallel with the acceleration.

**Figure 2.**
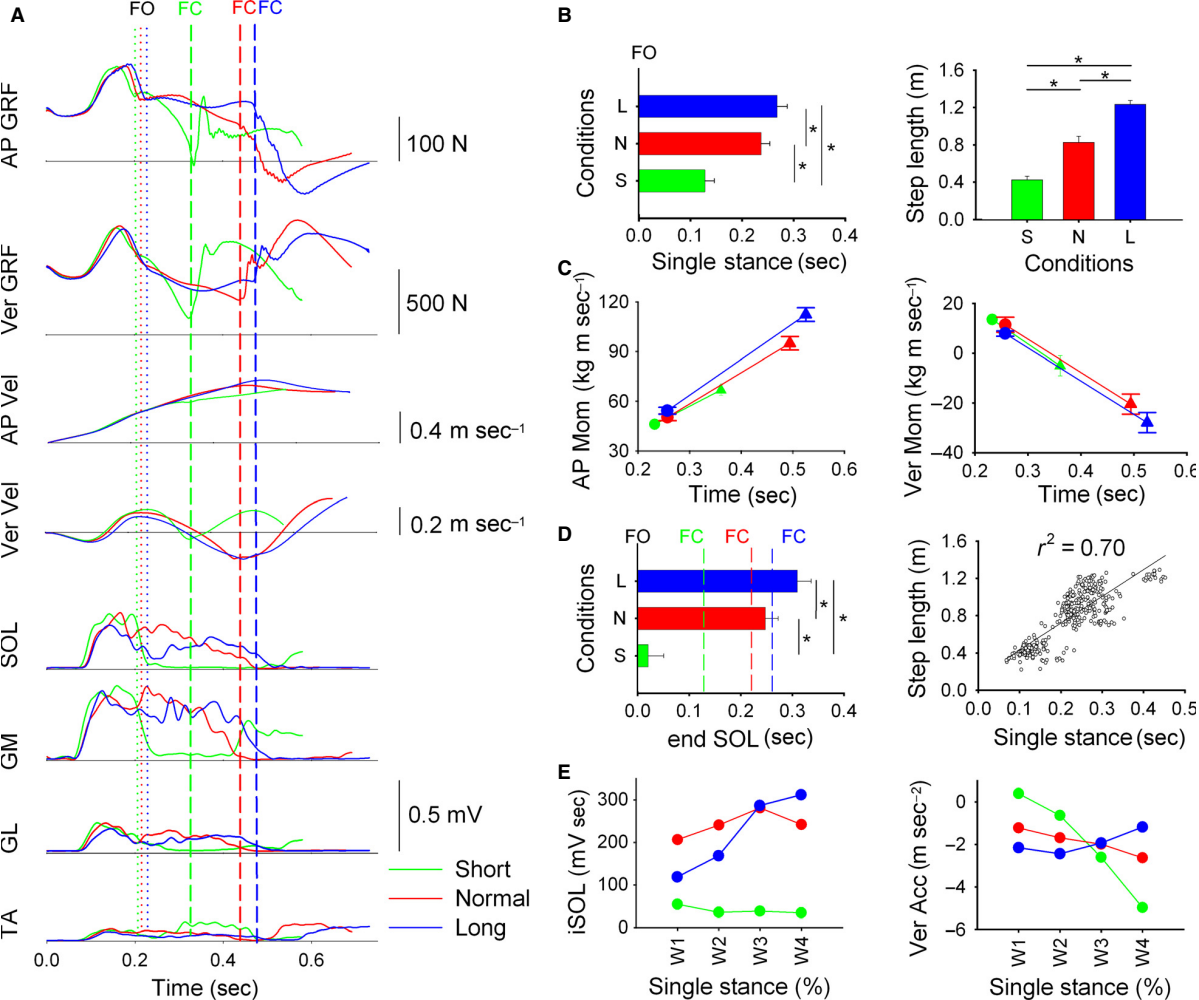
Balance recovery. (A) shows the time‐course of balance‐recovery variables in a representative subject (each trace is the average of 12 trials). Same layout as in Figure 1. In (B, left) the horizontal bars indicate the grand mean ± SD of duration of single stance. In (B, right) the bars depicts the grand mean ± SD of step length. In (C, left) the plot depicts the grand mean ± SD of CoM AP momentum at FO (circles) and FC (triangles). In (C, right) the plot depicts the grand mean ± SD of CoM Ver momentum at FO (circles), and FC (triangles). In (D, left) the horizontal bars are the grand mean ± SD of the end of activity of the EMG activity of SOL with respect to FO (*t* = 0). The dashed lines indicate the grand mean of the time of FC. In (D, right) step length is plotted against the single‐stance duration (all trials collapsed). There is a strict correspondence between step length and the duration of single stance. The linear equation is: *y* = 2.87*x* + 0.14. (E) shows the average values of the level of EMG activity of SOL (left) and the average CoM vertical acceleration (right) measured in the four equal time‐windows, in which the single‐stance phase was divided. In BR, SOL EMG activity was very low during single stance for short steps, which caused CoM to accelerate quickly downwards. In normal steps, SOL EMG activity was high throughout single stance, which decelerated the CoM fall with respect to short steps where SOL EMG level was low. In long steps, SOL EMG was highest in late stance, which decelerates further CoM fall during this period.

To better display the EMG activity (Figs. 1, 2), the envelopes of SOL, GM, GL, and TA were calculated. To do so, the EMG signals were rectified and low‐pass filtered with a Butterworth third‐order low‐pass zero‐lag filter. The cut‐off frequency was set at 30 Hz. To quantify the duration of triceps surae activity, the onset and end of the SOL, GM, and GL EMG were detected. These time instants were carefully identified visually on the raw EMG traces of the three muscles and expressed relative to the time, at which gait (GI) or BR initiated (at *t*0 = 0.0 sec in the figures). We also wanted to understand how the activity of the triceps surae was modulated in amplitude during single stance. The triceps surae activity was quantified based on the rectified SOL, GM, and GL EMG signals, which were time‐integrated from FO until FC. The integrals were then divided by the duration of the muscle activity to obtain the mean level of activity. In GI, the amplitude of TA activity was calculated by integrating the rectified EMG from *t*0 until FO. To further specify triceps EMG activity, we divided the single‐stance period in four equal‐duration intervals: early (0–25%), early‐mid (25–50%), midlate (50–75%), and late (75–100%) stance. Mean levels of SOL, GM, and GL EMG were then computed in each interval. The average vertical acceleration was also computed in each time window. A positive acceleration indicates that the CoM fall is being actively braked. Acceleration was therefore used in order to quantify the vertical braking action of CoM.

### Statistics

Analyses were performed with the SPSS software (SPSS 20.0, Chicago, IL). One‐way repeated‐measure analysis of variance (ANOVA) was performed across step‐length conditions in BR and across walking velocities in GI for each of the outcome variables: step length, CoM AP position at FC, CoM AP and Ver momentum at FO and FC, average EMG amplitude of SOL, GM, and GL during the single stance, average EMG amplitude of TA prior to single stance in GI, and finally CoM‐CoP gap and disequilibrium torque at FC. Two‐way ANOVA separately assessed the differences in the end of the EMG activity of SOL, GM, and GL. The first categorical factor was step length in BR and walking velocity in GI. The second factor was muscle (SOL, GM, and GL). Repeated‐measure ANOVA was used to assess differences in the average SOL, GM, and GL activity in addition to the average vertical acceleration of CoM in the four time windows, in which the single stance was divided. As in the one‐way ANOVA, the categorical factor was step length in BR and walking velocity in GI. The repeated‐measures were the four time‐windows. Post‐hoc tests were performed using the Bonferroni corrected paired *t*‐tests. The *P* value was set at 0.05. The linear relationship between the CoM‐CoP gap and CoM AP velocity was assessed using Pearson's *r*^2^ coefficient of determination.

## Results

This section is divided into three main parts. In the first two parts, we provide the results describing the triceps surae activity in relation to the two global kinematic variables of gait: cadence for GI and step length for BR. In the last part, we describe how propulsion is determined by modulating the disequilibrium torque via control of triceps activity in both the GI and BR protocol. In each part, qualitative data of a representative subject are followed by quantitative data, that is, grand means and standard deviations from all subjects. The result section only reports data measured during the execution of the first step, that is, from *t*0 till FC.

### GI: the duration of triceps activity determines cadence

Subjects were asked to initiate gait at different speeds without changing step length. For a fixed step length, cadence (proportional to the duration of single stance) must increase when walking faster and decrease when walking slower. So when walking slowly, we expected subjects to increase the duration of triceps activity, which would in turn increase single‐stance duration. The reverse should occur when walking fast. Figure 1A shows the time‐profiles of GRF and CoM velocity in AP and Ver directions and the envelopes of the EMG of SOL, GM, GL, and TA of the stance leg of a representative subject (each trace is the mean of 12 trials) under all GI conditions (green, red ,and blue indicate slow, normal, and fast walking, respectively). GRF traces are divided into double‐ (from *t*0 to FO) and single‐stance phase (from FO to FC). The disequilibrium torque is initiated by SOL silence and TA activation, both shifting the CoP backwards (Carlsöö 1966; Nardone and Schieppati 1988; Crenna and Frigo 1991; Jian et al. 1993; Lepers and Brenière 1995). It has been previously reported that TA activity increases when subjects initiate gait faster, in order to increase the momentum of the CoM prior to single stance (Lepers and Brenière 1995). In this experiment as well, TA increased its activity as subjects walked faster (Fig. 1A, bottom traces).

In the single‐stance phase, AP GRF increased and reached its maximum value slightly after FC. In the slow and normal condition, AP GRF showed a tendency to plateau before increasing toward late stance. In the fast condition, it continued rising until mid stance and increased further in late stance. At FC, the AP GRF peak was lowest when the subject walked slowly and highest when subjects walked faster. In all conditions, Ver GRF fell below bodyweight, reached a minimum value around mid stance and increased beyond bodyweight in late stance. Ver GRF value below bodyweight indicates that CoM is accelerating downwards, while Ver GRF above bodyweight reflects an upward CoM acceleration, or, in other terms, it indicates that the fall of CoM is being braked. AP CoM velocity increased in a parabolic fashion for all velocities and no difference was present in the shape of the traces across the three tasks before each respective FC. Ver velocity curves show that following FO CoM falls until mid stance, it then increases to reach a value around zero at FC. The minimum value of Ver CoM velocity is lowest during slow walking and highest during fast walking.

#### Duration of single stance and walking velocity

There was an effect of walking velocity on the duration of the single stance (one‐way ANOVA, *F*(2, 30) = 15.7, *P *<**0.001). The duration of the single stance decreased when walking faster and increased in slow walking (post‐hoc, *P *<**0.05 for all comparisons). In Figure 1B (left) the grand mean and standard deviations of the duration of the single stance (the length of the bars) is represented with respect to the grand mean of FO (origin of the abscissa). Figure 1B (right) shows the grand means and standard deviations of step length. As expected, walking velocity had no effect on step length (one‐way ANOVA, *F*(2, 30) = 0.18, *P *=**0.8).

#### AP and Ver momentum of CoM

In Figure 1C (left), the grand means and standard deviations of AP momentum of CoM at the time instant of FO (circles) and of FC (triangles) are shown. At foot off, there was a difference in CoM AP momentum (one‐way ANOVA, *F*(2, 30) = 9.2, *P *<**0.001). Momentum was not different between slow and normal walking (post‐hoc, *P* = 0.41), but increased in fast walking with respect to normal and slow speed (*P *<**0.05, for both comparisons). ANOVA showed a significant effect of walking velocity on TA activity just prior to single stance, that is, from *t*0 to FO (*F*(2, 30) = 8.32, *P *<**0.01). The post‐hoc test showed that the average TA EMG amplitude prior to FO was highest during fast walking (*P *<**0.05). One‐way ANOVA showed that the instant of FO was also slightly different between the three conditions. FO occurred earlier at normal speed than at fast and at slow walking momentum (post‐hoc, *P *<**0.05). At FC, there was a significant difference in CoM AP momentum between conditions (one‐way ANOVA, *F*(2, 30) = 19.2, *P *<**0.001). At FC, CoM AP momentum was highest for the fast condition and lowest during the slow condition (post‐hoc, *P *<**0.05).

In Figure 1C (right), the grand means and standard deviations of Ver momentum of CoM at the time of FO, at its minimum value and at FC are shown. The abscissa in the graph indicates the instant of FO (circles), of minimum value of Ver momentum (squares), and of FC (triangles). At foot off, there was a difference in CoM AP momentum (one‐way ANOVA, *F*(2, 30) = 10.5, *P *<**0.001). Momentum was not different between slow and normal walking (post‐hoc, *P* = 0.41), but increased in fast walking with respect to normal and slow speed (*P *<**0.05, for both comparisons). For the minimum value of CoM Ver momentum, ANOVA showed a difference between all conditions (*F*(2, 30) = 10.2, *P *<**0.001). The minimum value was lowest for slow walking and highest for fast walking. Finally, the vertical momentum of CoM at FC was not different between walking velocity conditions (one‐way ANOVA, *F*(2, 30) = 0.8, *P *=**0.4).

#### Duration of single stance and time‐course of triceps activity

Single stance was dominated by triceps surae activity. The duration of the activity and the duration of the single stance increased in the slow‐ and decreased in the fast‐velocity condition. For each velocity, two‐way ANOVA showed that the end of EMG activity was not different across the individual muscles (*F*(2, 30) = 0.12, *P *=**0.88), but revealed a significant effect of walking speed on the end of EMG activity (*F*(2, 60) = 69.4, *P *<**0.001). Grand means and standard deviations with respect to FO are shown in Figure 1D (left). The post‐hoc test indicated that the end of triceps surae EMG activity was anticipated for fast walking and was delayed for slow walking with respect to normal condition (*P *<**0.001, for all comparisons). As the temporal behavior of the three muscles was similar, then for clarity only grand means and standard deviations of SOL are provided in Figure 1D (left). The linear relationship between the end of SOL activity and the duration of the single stance had a coefficient of determination (*r*^2^ = 0.68) indicating a strong association between the two variables (Fig. 2D, right). The obtained linear equation was *y* = 0.49*x* + 0.14.

#### EMG activity and vertical braking action of CoM

To further investigate the modulation of triceps activity in the control of Ver acceleration, we calculated the average level of triceps EMG and Ver acceleration in four equal‐duration time‐windows of single stance (W1–W4 in Fig. 1E). Repeated‐measures ANOVA showed a difference in the EMG level of each muscle for the three walking velocities (SOL, *F*(2, 30) = 4.6, *P *<**0.05; GM, *F*(2, 30) = 9.7, *P *<**0.05; GL, *F*(2, 30) = 12.4, *P *<**0.001). Post‐hoc test showed an increase in EMG level when walking faster (*P *<**0.01, for all comparisons). The post‐hoc test indicated that triceps activity increased progressively from one time‐window to the next in each of the three muscles for each of the walking velocity conditions (*P *<**0.05). In W1, the post‐hoc showed no effect of walking velocity on triceps activity. In W2, triceps EMG level increased only in the fast condition (*P *<**0.01). In mid stance to late stance, triceps EMG level was highest in fast and lowest in slow walking in W3 (*P *<**0.05), while in W4 EMG level was lower in slow walking with respect to the normal condition and highest in the fast condition (*P *<**0.05). As the results obtained for each of muscle were superimposable, then for clarity only data of SOL EMG are shown in Figure 1E (left panel).

Repeated‐measure ANOVA also showed that Ver CoM acceleration was different in the four time‐windows (*F*(3, 40) = 24.5, *P *<**0.001, respectively). The CoM accelerated downwards during early and mid stance. It reverted upwards in mid to late stance and became positive, that is, vertical velocity decreased in late stance. In W1 and W2, Ver CoM acceleration was highest in fast walking (*P *<**0.05 for both time‐windows), but did not change between normal and slow walking. Ver CoM acceleration did not change in W3. In W4, Ver CoM acceleration was always positive, indicating that CoM vertical velocity is decreasing. The braking action on CoM was highest for fast and lowest for low walking (*P *<**0.01). This suggests that when CoM vertical velocity increases as a result of fast walking, the triceps activity in late stance augments in order to increase the braking action. The increase in the braking action decreases vertical momentum so that at FC CoM Ver velocity is unchanged between walking velocity conditions.

### BR: triceps surae activity determines step length

Figure 2A shows the mean (12 trials) time profiles of GRF and CoM velocity in AP and Ver directions, and the EMG of SOL, GM, GL, and TA envelopes of the stance leg of one representative subject in all balance‐recovery conditions (green, red, and blue indicate short, normal, and long steps, respectively). GRF traces are divided into double‐ (from *t*0 to FO) and single‐stance phase (from *t*0 to FC).

For a brief period (less than 250 msec) following the release of the cable, both feet were in contact with the floor. CoM was initially positioned largely ahead of the CoP due to the inclination of the body, and the release of the cable caused the gravitational torque to have a high value due to the presence of a large CoM‐CoP gap and the BW of the subject. The torque caused CoM to fall rapidly for around 50 msec. The fall was followed by a reaction phase characterized by upward and forward increase in GRF and short‐latency activation of triceps surae (Do et al. 1990). The upward surge in GRF caused the CoM to accelerate upwards, during which period subjects recovered a more vertical orientation of the body. In this time period, no major difference in GRF and EMG can be observed between all step‐length conditions during double stance, as previously shown by Do et al. (1982).

Single‐stance features were instead clearly different between short‐, normal‐, and long‐step conditions. In the normal condition, that is, when subjects had no instruction about step length, both AP and Ver GRF decreased until FC. EMG activity in all three heads of the triceps was large in early stance and decreased throughout single stance until ending at about the instant of FC. When executing short steps, GRF decreased rapidly reflecting a high CoM downward acceleration. This caused the swing foot to touch the floor early resulting in the execution of the short step. Triceps activity ended just slightly after FO. The lack of triceps EMG activity in the stance phase of short steps reduces dramatically the body‐support, causing the high downward acceleration of CoM. In normal steps, triceps EMG was present throughout single stance, causing CoM to reduce downward acceleration, as reflected by the decrease in Ver GRF, with respect to the short‐step condition. For long steps, the subject prolonged the duration of the triceps EMG burst, thus delaying the instant of FC. The EMG amplitude was lower in early stance and increased toward midlate stance. GRF also decreased in early stance and increased in late stance. During single stance, AP velocity increased in an almost linear fashion. The time profile of AP velocity was similar during each step‐length condition until FC that mechanically braked forward progression. CoM Ver velocity decreased after FO. In short step, Ver velocity reached a slightly negative value, since duration of single stance was short. During normal step, Ver velocity decreased throughout single stance. In long step, Ver velocity decreased too. However, CoM accelerated faster downwards in middle stance with respect to normal step, but then decelerated so that the instantaneous value of the CoM Ver velocity at FC did not change between the normal‐ and the long‐step condition.

#### Duration of single stance and step length

Grand means and standard deviations of the duration of single stance are shown in Figure 2B (left). Step length had a significant effect on the duration of the single stance (one‐way ANOVA, *F*(2, 30) = 107.8, *P *<**0.001). The post‐hoc test indicated that subjects varied the single‐stance duration in order to execute steps of different lengths (*P *<**0.001, for all comparisons). In the right panel of Figure 2B, the means and standard deviation of step length at FC are shown. As expected, step length was different across conditions (*F*(2, 30) = 143.2, *P *<**0.001) (for all paired comparisons, *P *<**0.001).

#### CoM AP and Ver momentum

Figure 2C (left) shows the AP momentum of CoM at both FO and FC. The abscissa indicates the instant when FO (circles) and FC (triangles) occurred. At FO, there was no significant effect of step length on CoM AP momentum (one‐way ANOVA, *F*(2, 30) = 2.1, *P *=**0.14). The mean CoM AP momentum (all trials collapsed) was around 50 kg·m·sec^−1^ at FO, which is much higher than that obtained normally in GI, as the disequilibrium torque due to the initial CoM‐CoP gap linked to body inclination was responsible for the propulsion in the period preceding the step execution. At FC, ANOVA showed a significant effect of step length on CoM AP momentum (*F*(2, 30) = 42.4, *P *<**0.001. CoM AP momentum was highest for long steps and lowest for short steps (post‐hoc, *P *<**0.001).

Figure 2C (right) shows the Ver momentum of CoM at both FO and FC. At FO, there was no significant effect of step length on CoM Ver momentum (*F*(2, 30) = 1.9, *P *=**0.16). The mean CoM Ver momentum at FO was around 10.5 kg·m·sec^−1^ (all trials collapsed). At FC, ANOVA showed a significant effect of step length on CoM Ver momentum (*F*(2, 30) = 29.44, *P *<**0.001). Post‐hoc test showed that CoM Ver momentum at foot contact was lower in the short steps compared to normal and long steps (*P *<**0.001, for both comparisons). However, Ver CoM momentum did not change between normal and long steps (*P *=**0.17).

#### Triceps temporal modulation and step length

Two‐way ANOVA showed that the end of EMG activity was not different across muscles (*F*(2, 30) = 0.21, *P *=**0.97), but there was a significant effect of step length on end of EMG (*F*(2, 60) = 800.4, *P *<**0.001). The end of triceps EMG activity was anticipated for short steps and delayed for long steps with respect to normal condition (*P *<**0.001, for all comparisons). As the temporal behavior of the three muscles was similar, only the grand means and standard deviations of the end of SOL activity are provided in Figure 2D (left). The end of triceps activity occurred just shortly after FO in short steps, slightly following FC in the normal condition and well after FC for long steps.

A high coefficient of determination was found between the end of SOL activity and the duration of single stance in normal and long steps (*r*^2^ = 0.91) (not shown in Fig. 2). The obtained linear equation was: *y* = 2.1*x* − 0.01. In the short‐step condition, the initial inclination of the subjects forced them to react quickly to stop the EMG activity as early as possible in order to allow premature contact with the floor. A high coefficient of determination between step‐length and single‐stance duration was obtained (*r*^2^ = 0.70) (Fig. 2D right, all trials collapsed). The obtained equation was: *y* = 2.87*x* + 0.14.

#### EMG activity and vertical acceleration of CoM

In the BR experiment, the CoM is initially placed away from the CoP and at a lower position than in erect posture. During the balance‐recovery step, this condition constrains the CNS to compensate for the imposed gap and check the vertical force by triceps surae activity. To investigate how this is done, we examined the triceps EMG level and Ver CoM acceleration in the four time‐windows of single stance. Repeated‐measures ANOVA showed a difference in the EMG area of each muscle for the different step lengths (SOL, *F*(2, 30) = 36.2, *P *<**0.001; GM, *F*(2, 30) = 9.4, *P *<**0.01; GL, *F*(2, 30) = 37.2, *P *<**0.001). For short steps, SOL activity decreased rapidly, which caused a rapid downward acceleration of CoM. For the normal steps, SOL activity increased until midlate stance and then decreased in late stance. CoM downward acceleration increased but at much lower rate that in short steps. For long steps, triceps EMG level was low in early to mid stance and increased in mid to late stance. Post‐hoc test showed that EMG level was always significantly different (*P *<**0.001) in all time‐windows, except in W3 between normal and long steps (*P *<**0.01). For short steps, triceps EMG activity was down to base level during single stance. Otherwise, triceps EMG level were highest in normal steps in early to mid stance with respect to long steps. However, In W4, triceps EMG level was highest in long steps with respect to normal steps. As the results obtained for each of muscle are similar, then for clarity only results of SOL EMG level are shown in Figure 1E (left panel).

Repeated‐measure ANOVA also showed that Ver acceleration was different in the four time‐windows (*F*(3, 40) = 103.5, *P *<**0.001). Ver acceleration was always negative in all time‐windows and for all step‐length conditions. Post‐hoc test showed that Ver acceleration was always different (*P *<**0.001) in all time‐windows, except in W3 between normal and long steps (*P *<**0.01). In short steps, the lack of triceps activity during single stance, cause CoM to quickly accelerate downwards and establish early contact with the ground. In normal steps, the presence of triceps activity decreased downward acceleration with respect to short steps where no activity was present. In long step, the surge of triceps activity in late stance decreased further the downward acceleration of CoM.

### Disequilibrium torque in GI and BR

**Figure 3.**
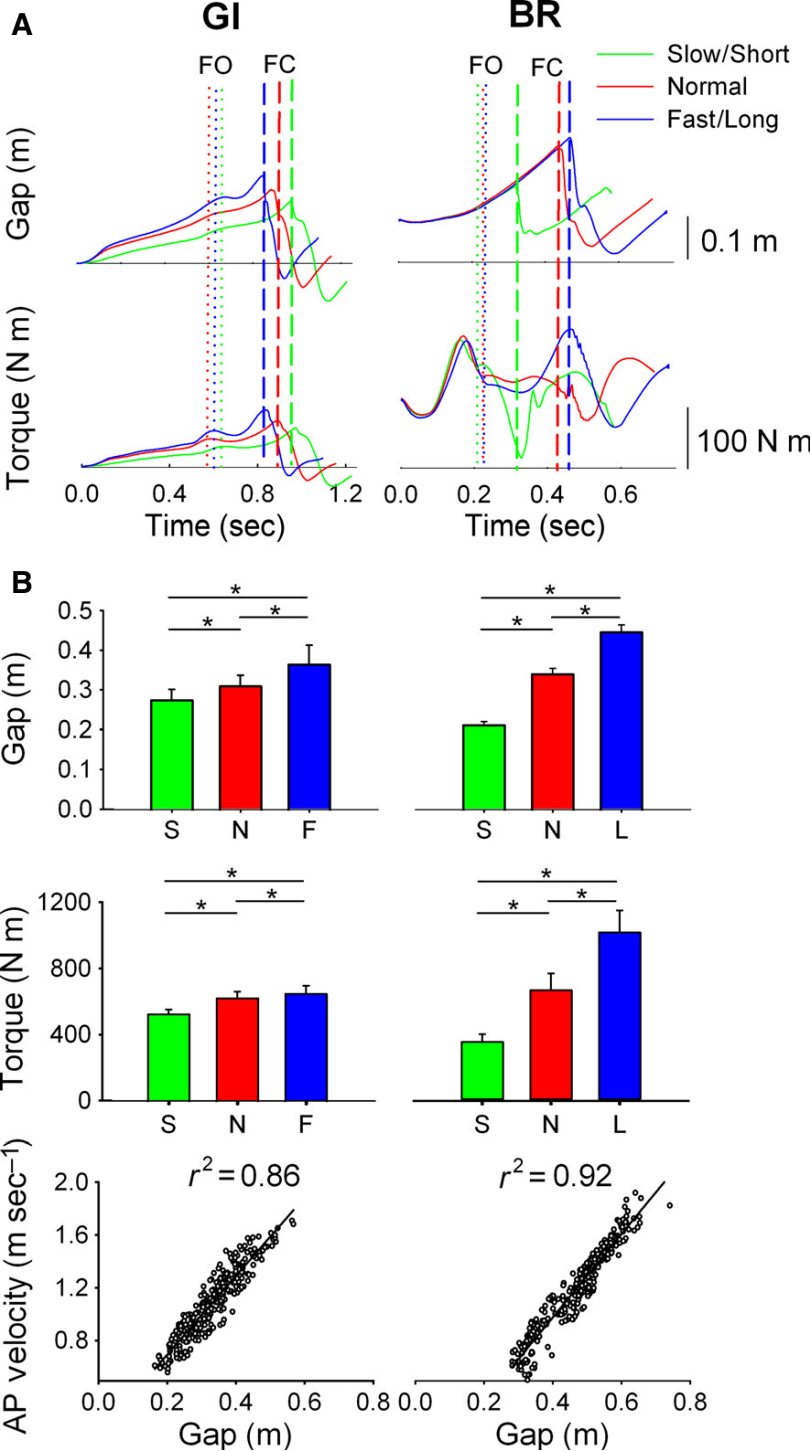
Disequilibrium torque. This figure shows the results obtained during GI (left) and BR (right). In (A) the mean (average of 12 trials) time‐courses of CoM‐CoP gap and of disequilibrium torque of a representative subject are shown. Same layout as Figure 1 In (B, upper panel) the bars show the grand mean ± SD of the CoM‐CoP gap at FC. In (B, middle panel) the bars show the grand mean ± SD of the disequilibrium torque at FC. (B, lower panel) shows the position of the CoM in the sagittal plane (CoM‐CoP gap) plotted against the CoM velocity (all trials collapsed). The linear equations obtained for GI and BR are *y* = 2.97 + 0.1 and *y* = 2.95*x* − 0.13. The high coefficient of determination and the equal slopes emphasizes the role of the CoM‐CoP gap in equally setting the velocity of the walking body regardless of the imposed task.

As shown previously in Figure 1A, the preparatory phase (from *t*0 to FO) of GI was marked by SOL silence and TA activation. This shifted the CoP backwards creating a gap between the CoM and CoP in the sagittal plane that initiated the disequilibrium torque. As stated earlier, TA activity increased in GI when walking faster, thereby increasing the backward shift of CoP, so that gap and torque reached greater instantaneous amplitudes at FO. In the single‐stance phase, CoM traveled further away from CoP causing gap and torque to build up. In late stance, the gap and torque showed a steep increase (Fig. 3A, left). CoM‐CoP gap at FC was different between the three walking velocity conditions (one‐way ANOVA, *F*(2, 30) = 8.9, *P *<**0.001). It was smallest in slow steps and highest in fast steps (*P *<**0.05, for all comparisons). Mean values of the gap are reported in Figure 3B (upper left). At FC, Ver GRF (that is the other component of the torque) was not different between the three walking velocities (*F*(2, 30) = 0.05, *P *=**0.9) (not shown). Mean and standard deviation of disequilibrium torque are shown in Figure 3B (middle left). The disequilibrium torque at FC was different between walking velocity conditions (*F*(2, 30) = 31.8, *P *<**0.001). It was smaller in short steps and higher in long steps (*P *<**0.01, for all comparisons). Figure 3B (bottom left) shows a scatter plot of the CoM‐CoP gap against the CoM velocity at FC (all trials of all subjects collapsed). Gap and walking velocity at FC had a strong linear relationship (*y* = 2.97*x* + 0.1; *r*^2^ = 0.86).

In BR, prior to the release of the cable, the AP position of CoM is located around 17 cm forward to that of CoP. This caused the disequilibrium torque to increase very rapidly at the time of the release. During single stance, the CoM‐CoP gap increased in a similar fashion in all three step‐length conditions, because in BR the CoM AP velocity was unchanged at FO. However, the time‐profile of Ver GRF changed considerably between conditions, which caused considerable changes in the time‐profile of the disequilibrium torque (Fig. 3A, right panel). One‐way ANOVA showed that CoM‐CoP gap at FC was different between the three step‐length conditions (*F*(2, 30) = 29.6, *P *<**0.001). Mean values of the gap are shown in Figure 3B (upper right panel). The post‐hoc test showed that gap was smaller in short and higher in long steps, respectively (*P *<**0.01, for all comparisons). One‐way ANOVA showed that at foot contact, Ver GRF was significantly different between the three walking velocity conditions (*F*(2, 30) = 17.9, *P *<**0.001) (not shown), its amplitude at FC being lowest in short steps and highest in long steps. Finally, one‐way ANOVA showed that disequilibrium torque at FC was different between walking velocity conditions (*F*(2, 30) = 27.8, *P *<**0.001). The post‐hoc test showed that torque was smaller in short steps and higher in long steps (*P *<**0.01, for all comparisons). Grand mean and standard deviation of disequilibrium torque are shown in Figure 3B (middle right). Gap and walking velocity at FC had a strong linear relationship (*r*^2^ = 0.92) (bottom right). The obtained linear equation was *y* = 2.95*x* − 0.13.

## Discussion

### Rationale of the experiment

It has been proven long time ago that the changes in potential and kinetic energy during human gait are conveniently in opposite phase “as in a rolling egg” (Cavagna and Margaria 1966). This allows the pull of gravity to generate a disequilibrium torque, which in turn produces propulsive force (Cavagna et al. 1976; Cavagna and Franzetti 1986; Honeine et al. 2013) that ultimately determines the velocity of progression.

In our daily life, we walk at different velocities and are capable of exercising all combinations of step length and cadence to produce a certain velocity (Alexander 1984; Nilsson et al. 1985; Stoquart et al. 2008; Ivanenko et al. 2011; Leurs et al. 2011). As yet, little insight is available on how the constant pull of gravity is exploited as to produce different walking velocities. Obviously, modulation of walking velocity occurs through control over lower limb muscle activity. Indeed, progression speed and triceps surae activity have been shown to covary (Winter 1983; Hof et al. 2002; Den Otter et al. 2004; Courtine et al. 2006). However, in a previous paper, we provided evidence, by adding a load to the body, that the main function of triceps surae during walking is to decelerate the CoM fall and not to propel it forwards (Honeine et al. 2013). In the present study, we hypothesized that, during single stance, triceps surae can set global gait kinematics and kinetics by appropriately tailoring its decelerating action on the pull of gravity.

In gait, the two global kinematic variables, step length and duration of single stance, are strongly linked (Murray et al. 1964; Öberg et al. 1993). In this study, we needed to devise an experiment that allowed testing how triceps modulation can generate different combinations of step length and cadence to obtain a wide range of walking velocities. We decided to use two experimental protocols. In the first protocol (GI), we studied how triceps surae activation can modulate single‐stance duration. In the second (BR), we studied how triceps surae activation varies step length. Combining both results obtained in GI and BR allowed us to get insight into the link between muscle activation and walking.

The GI and BR protocols are well‐documented in the literature (Carlsöö 1966; Brenière et al. 1981; Do et al. 1982, 1990; Brenière and Do 1991; Pai and Patton 1997; Aftab et al. 2012). In GI, we asked subjects to initiate gait keeping step length constant (Brenière and Do 1991; MacDougall and Moore 2005) in different trials, performed at slow, spontaneous, or fast velocity. Thus, according to the formula *V* = *L* × *C*, subjects modified cadence (an index of which was the duration of the single stance). In BR, subjects were initially inclined by means of a cable, the release of which provoked an automatic step at a constant initial velocity of CoM progression. Subjects recovered balance by executing short, normal, and long steps.

### Modulation of triceps activity determines the global kinematics of gait

In human gait, muscle activity in the swing leg is present only at FO and FC (Basmajian 1976), that is, the swing leg moves under the effect of gravity and body's kinetic energy in a quasi‐ballistic mode (Mochon and McMahon 1980). Consequently, the CNS sets step length and duration of single stance by controlling the CoM momentum of the body by means of modulating the activity of the muscles of the stance leg.

In both protocols, the duration of the single stance proved to be highly correlated with the duration of triceps activity. More precisely, the duration of the triceps activity was shortened in order to reduce the duration of the single stance, and lengthened in order to increase it. Hence, by controlling the duration of triceps activity, the CNS determines the duration of the single stance and thus cadence. On the other hand, by modulating the amplitude of the CoM momentum during single stance, the CNS controls the distance traveled by the falling body before the swing leg establishes contact with the floor. In other terms, a particular amount of CoM momentum occurring during a definite duration of single stance should result in a specific step length. The BR protocol provided insight into how duration of single stance and CoM momentum are modulated by triceps activity to set step length. Firstly, the duration of the single stance was highly correlated with step length. Secondly, as the CoM velocity at FO was equal across step‐length conditions, CoM momentum was solely modulated by means of varying the level activity of triceps surae EMG. In short steps, triceps surae was almost silent during single stance. In this special condition, the inverted pendulum is driven solely by gravity. CoM accelerates quickly downwards and the swing leg establishes premature contact with the floor. In normal‐length steps, triceps surae activity builds up and cushions the fall of CoM. This reduces the downward acceleration of CoM and at the same time allows it to travel further away from the stance foot before FC. In long steps, triceps activity is initially low in early and in mid stance. This allows the CoM to accelerate quickly forwards and downwards. In mid stance to late stance, the triceps surae activity increases and reduces the CoM downward acceleration. Consequently, the foot contact is delayed, while the instantaneous Ver velocity of CoM at FC is unchanged between normal and long steps.

### Disequilibrium torque and propulsive force are modulated via the CoM‐CoP gap

From a mechanical point of view, walking velocity is the result of applying propulsive force against the ground via the stance foot. In Honeine et al. (2013), we showed that propulsive force is the result of the disequilibrium torque. The torque is the product of the CoM‐CoP gap and the vertical force acting on the CoM. The present study shows that by controlling the gap, triceps activity modulates the disequilibrium torque and so the propulsive force and walking velocity. Controlling the duration of the single stance and CoM momentum allows the CNS to determine the position of the CoM relative to CoP, that is, the CoM‐CoP gap. In the fast‐walking condition of GI, the high momentum of CoM at FO allows it to travel further away from CoP than in the normal condition. The opposite occurs during slow walking. In BR, where CoM position and step length are highly linked together due to biomechanical constraints, CoM‐CoP gap increases progressively as step length increases. Controlling CoM‐CoP gap modulates the disequilibrium torque, thus propulsive force and walking velocity. Indeed, the CoM‐CoP gap was highly correlated with walking velocity at FC in both protocols. Interestingly, the slopes of the linear equations of walking velocity plotted as a function of the gap were very similar in the two different protocols (2.97 for GI and 2.95 for BR). This strongly suggests that the amount of increase in the CoM‐CoP gap when velocity increases is the same regardless of which kinematic variable is being modulated (i.e., cadence or step length). We would like to underline the fact that two different populations performed the GI and BR experiments. Therefore, the difference in bodyweight that is a factor in determining the disequilibrium torque should have resulted in the difference in the intercept of the two linear equations obtained in the two experiments. We, thus, assume that if the same population performed both experiments, a certain CoM‐CoP gap would have always resulted in the same walking velocity in both protocols. Further experimentation is required to ascertain this fact.

### Braking action of CoM and push off

In gait, triceps EMG increases alongside the AP and Ver GRF in late stance. The rise in GRF is termed “push off”. Many authors have associated the increase in the triceps surae EMG in late stance with the push off (Winter 1983; Anderson and Pandy 2001; Neptune et al. 2001). On the contrary, in Honeine et al. (2013), we showed that triceps EMG does not increase when subjects initiate gait with a load with respect to no‐load at the same walking velocity, in spite of the obligatory increase in propulsive force due to the increase in bodyweight. The vertical braking action of CoM did not change, either, with the added load. The braking action has been previously reported by Chong et al. (2009) and Chastan et al. (2010). It is quantified by the amount by which Ver velocity of CoM is reduced in late stance. However, in Honeine et al. (2013), when subject walked faster both the braking action and triceps activity increased. In the present study, we provide proof that the reason of the surge in triceps activity in faster walking is due to the increased requirement of the CoM vertical braking action.

In the GI experiment, CoM vertical momentum increased as a function of walking speed (Fig. 1C, left panel) and caused by a rise in TA activity prior to single stance (Nardone and Schieppati 1988; Lepers and Brenière 1995). Therefore, around middle stance the downward velocity of CoM was highest in fast walking. Thus, a higher vertical momentum requires the application of a stronger upward force in late stance to brake the CoM fall. The increase in upward force is done by enhancing triceps activity. Consequently, the CoM Ver velocity at FC is unchanged regardless of the walking speed condition. Verdini et al. (2006) suggested that reducing vertical acceleration of the falling CoM allows for a softer touchdown of the swing leg with the floor for a smoother step‐to‐step transition in the subsequent double‐stance phase. Kuo (2007) explained that the step‐to‐step transition is dependent upon the vertical force applied by the stance leg and by the swing leg as it touches the ground. He inferred that the forces provided by both legs help redirect the CoM upwards during double stance. We suggest that, by braking the fall of CoM, the CNS would also modulate the force produced by the swing leg as it impacts the floor and thus control the redirection of the CoM during step‐to‐step transition.

### General considerations and limitations

Donelan and Kram (1997), Cavagna et al. (2000) and Sylos‐Labini et al. (2013) all had previously shown that walking in a reduced gravity environment changes significantly gait kinematics. In other terms, kinematics of walking rely strongly on the vertical force acting on CoM. In this study, under constant gravitational attraction, the vertical force aimed to produce different gait kinematics is modulated by triceps surae activation, which counteracts the downward pull of gravity. During single stance, humans are always in a state of disequilibrium and it is the torque caused by the disequilibrium that generates propulsion. By appropriately opposing the pull of gravity, CNS is capable of modulating the disequilibrium torque and of producing all combinations of step length and cadence merely through control of triceps activity of the stance leg.

Recent findings have given evidence that motor cortex and corticospinal tract contribute directly to the muscle activity observed in steady‐state treadmill walking (Petersen et al. 2012). The CNS would therefore be able to select and implement each combination of cadence and step length to produce a given walking velocity. In this study, we used the GI and BR protocols to prove our hypothesis. In a sense, in our case, deliberate intervention of the higher brain centers engaging corticospinal control was unavoidable and warranted. On the other hand, a reflex contribution to this process should be acknowledged because sensory feedback plays a critical role exactly in the adaptation of activation of extensor motoneurons during locomotion (Sinkjaer et al. 2000; Tokuno et al. 2007; Van Doornik et al. 2011). In addition to the facilitatory inflow from primary and secondary muscle spindle fibers (Mazzaro et al. 2005), a relevant feedback would come from the load receptors present in plantar‐flexors, which are known to exert a major action in human locomotion (Dietz and Duysens 2000). Indeed, the fall of the CoM during single stance partly unloads the Golgi tendon organs in the Achilles tendon. Thus, the feedback signal via the group Ib afferent fibers would decrease alpha‐motoneuron inhibition and increase the firing rate of the triceps motor units during the braking action, together with the enhanced facilitatory spindle input, therefore ultimately opposing the fall of the CoM.

In spite of the obvious differences between GI or BR and spontaneous steady‐state walking, we would note that in GI and BR and steady‐state gait, the CoM rotates around the stance foot during step execution for a considerable range of walking velocities (Cavagna and Franzetti 1986). We believe that, within that range, the conclusions obtained in our experiments from the parallel analysis of the spatiotemporal EMG and the biomechanical constraints can be largely extrapolated to steady‐state walking. It is also to be noted here that when executing very long steps, the body stops acting as a controlled inverted pendulum (Cavagna and Franzetti 1986). Indeed, the speed at which our subjects performed the long steps in BR did sometimes reach that at the transition from walking to running (Cavagna and Kaneko 1977; Thorstensson and Roberthson 1987; Diedrich and Warren 1995; Hreljac et al. 2005). Thus, the results discussed in this study are valid within a wide range of walking velocities, yet far from those occurring close to transition to running.

In our hands, no significant differences between the onset and end of activity of the three heads of the triceps surae were found. They seem to obey the same motor command that has been traced to their motor pools in the spinal cord (Cappellini et al. 2010). Notably, the central pattern generator (CPG) for locomotion commonly recruits the three components of the triceps (Dimitrijevic et al. 1998; Lacquaniti et al. 2012). However, one limitation of this study is that we are not in the position of comparing the force contribution of each of the individual muscles. The three muscles plantar‐flex the ankle, but they can have antagonistic behavior across the knee (Van Ingen Schenau et al. 1987). During responses to postural perturbation by displacement of the support base, the three heads can behave differently (Nardone et al. 1990). On the other hand, even though authors do not agree on the exact role of each individual muscle, everybody agrees that all three heads of triceps contribute in body support (Anderson and Pandy 2001; Neptune et al. 2001; Francis et al. 2013).

## Conclusion

Humans have evolved to take advantage of the torque driven by gravity to propel themselves. When we learn to walk, the spinal circuits ultimately driving the triceps motoneurons are gradually conquered by the developing descending tracts (Musienko et al. 2011), which learn “to talk to the spinal cord in a language that it can understand, determined by its pre‐existing circuits” (Matthews 1995). Such process makes bipedal walking possible by making representation of gravity part of our brain activity (Papaxanthis et al. 2003; Ivanenko et al. 2006) and by exploiting gravity to produce propulsive force (Cavagna et al. 1976; Honeine et al. 2013). In this work, we show that modulation of triceps surae in amplitude and duration allows the CNS to perform all the possible combinations of step length and cadence. In the process, the disequilibrium is set and consequently walking velocity.

We believe that such capacity worsens when the interaction between brain, CPG, interneurons and motoneurons, and sensory feedback fails. Gait is abnormal in peripheral neuropathy (e.g., Casasnovas et al. 2008; Wrobel and Najafi 2010; Nardone et al. 2014). CNS problems also produce dramatic effects, notably in stroke patients showing inconsistent step‐length asymmetries (Roerdink and Beek 2011), in Cerebral Palsy patients, where changes in muscle fiber properties together with altered reflex excitability (Berger et al. 1982) modify the stance phase of gait, and in Parkinsonian patients, who are unable to produce long steps, relying instead on short and frequent steps (De Nunzio et al. 2010; Nanhoe‐Mahabier et al. 2011). Interestingly, replacement of one lower limb by a prosthesis does not affect the speed of progression, regardless of whether the stance limb was prosthetic or not. Most likely, other muscles take over the control of the duration of the stance phase of gait and of the BM CoM momentum (Michel and Do 2002; Michel and Chong 2004; Wentink et al. 2013). Whether these subjects can still manage to select different stepping frequencies or step lengths has not received much attention to date (Roerdink et al. 2012).

## Acknowledgment

The authors are grateful to the Kremlin Bicêtre hospital for allowing the usage of their laboratory to perform the experiments.

## Conflict of Interest

None declared.
